# Insights into Structure-Activity Relationships of Somatostatin Analogs Containing Mesitylalanine

**DOI:** 10.3390/molecules181214564

**Published:** 2013-11-25

**Authors:** Pablo Martín-Gago, Eric Aragón, Marc Gomez-Caminals, Jimena Fernández-Carneado, Rosario Ramón, Pau Martin-Malpartida, Xavier Verdaguer, Pilar López-Ruiz, Begoña Colás, María Alicia Cortes, Berta Ponsati, Maria J. Macias, Antoni Riera

**Affiliations:** 1 Institute for Research in Biomedicine (IRB Barcelona) Baldiri Reixac, 10, Barcelona 08028, Spain; E-Mails: pablo.martin@irbbarcelona.org (P.M.-G.); eric.aragon@irbbarcelona.org (E.A.); rosario.ramon.albalate@gmail.com (R.R.); pau.martin@irbbarcelona.org (P.M.-M.); xavier.verdaguer@irbbarcelona.org (X.V.); 2 BCN Peptides S.A. Pol.Ind. Els Vinyets-Els Fogars, Sector II. Ctra. Comarcal 244, Km. 22, 08777 Sant Quintí de Mediona, Barcelona 08777, Spain; E-Mails: mgomez@bcnpeptides.com (M.G.-C.); jfernandez@bcnpeptides.com (J.F.-C.); bponsati@bcnpeptides.com (B.P.); 3 Departament de Química Orgànica, Universitat de Barcelona, Martí i Franqués, 1-11, Barcelona 08028, Spain; 4 Departamento de Bioquímica y Biología Molecular, Universidad de Alcalá de Henares, Facultad de Medicina, Madrid 28871, Spain; E-Mails: pilar.lopezruiz@uah.es (P.L.-R.); begona.colas@uah.es (B.C.); alicia.cortes@uah.es (M.A.C.); 5 Institució Catalana de Recerca i Estudis Avançats (ICREA), Passeig Lluis Companys, 23, Barcelona 08010, Spain

**Keywords:** somatostatin, drug design, peptidic hormones, non-covalent interactions, NMR, structure-activity relationships, conformational analysis

## Abstract

The non-natural amino acid mesitylalanine (2,4,6-trimethyl-L-phenylalanine; Msa) has an electron-richer and a more conformationally restricted side-chain than that of its natural phenylalanine counterpart. Taking these properties into account, we have synthesized ten somatostatin analogs containing Msa residues in different key positions to modify the intrinsic conformational flexibility of the natural hormone. We have measured the binding affinity of these analogs and correlated it with the main conformations they populate in solution. NMR and computational analysis revealed that analogs containing one Msa residue were conformationally more restricted than somatostatin under similar experimental conditions. Furthermore, we were able to characterize the presence of a hairpin at the pharmacophore region and a non-covalent interaction between aromatic residues 6 and 11. In all cases, the inclusion of a D-Trp in the eighth position further stabilized the main conformation. Some of these peptides bound selectively to one or two somatostatin receptors with similar or even higher affinity than the natural hormone. However, we also found that multiple incorporations of Msa residues increased the life span of the peptides in serum but with a loss of conformational rigidity and binding affinity.

## 1. Introduction

### 1.1. Somatostatin Analogs

**Figure 1:**
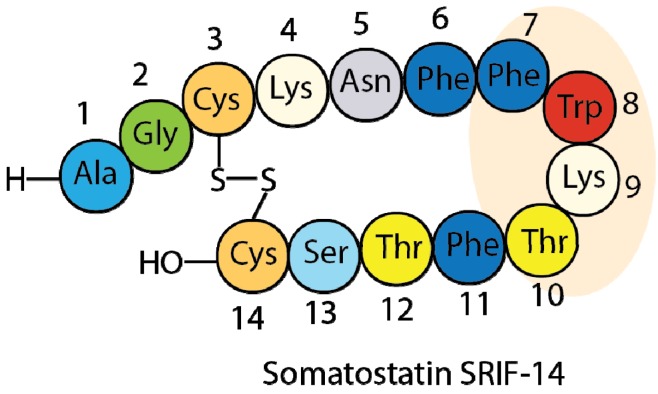
Amino acid sequence of somatostatin.

Site directed mutagenesis is a well-established method to modify the properties of proteins and peptides. These modifications normally include changes in hydrophobicity, stability, conformation and biological activity of the new molecules [1,2,3,4]. Somatostatin [5,6], also known as somatotropin release-inhibiting factor (SRIF-14) (Figure 1), is one of the most studied peptides due to its important biological properties. This hormone is produced in the hypothalamus and is involved in multiple biological functions, mediated by its direct interaction with at least five different G-protein coupled receptors, named SSTR1-5 [7,8]. The use of this hormone in several different clinical treatments (anti-secretory drug, growth hormone secretion disorders and endocrine tumor treatment) [9,10,11,12] reflects its pharmacological importance. However, its short half-life (2–3 min in plasma *in vivo*) and its low selectivity (high binding affinity towards all five different receptors) are the major disadvantages of natural SRIF as a drug.

**Figure 2:**
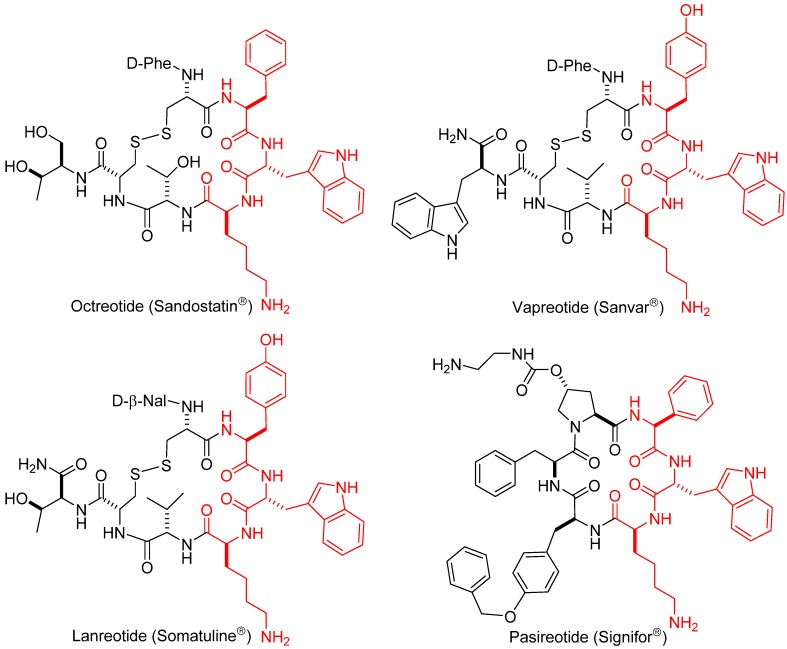
Amino acid sequences of the marketed short-ring analogs.

A myriad of SRIF analogs have been synthesized over the past few decades introducing modifications such as exchange and deletion of amino acids, ring size reduction, disulfide bridge modification, multiple N-methylation and site-specific PEGylation [13,14,15,16]. Several of those analogs displayed an improvement of different “drug-like” properties in comparison to somatostatin. A few synthetic analogs have reached the market: octreotide (Sandostatin^®^) [17,18] lanreotide (Somatuline^®^), vapreotide (Sanvar^®^) and pasireotide (Signifor^®^) [19] (Figure 2). These are octa- or hexapeptides, thus having a shorter and consequently less flexible ring than that of somatostatin. They are long-acting analogs with increased plasma stability and are highly selective against receptor SSTR2. To date, most of the research efforts in this field have focused on the design of new more conformationally restricted analogs with shorter rings (octreotide analogs, in fact), and on the improvement of the methodology to prepare them in an efficient and simple manner. The current solid-phase protocols have diminished the challenge of obtaining 14-residue’s peptides, and thus, our approach to prepare new analogs is based on synthesizing full-length SRIF-14 analogs with particular site-directed modifications [20,21]. Our aim is to improve the stability and receptor selectivity of natural somatostatin and to overcome the main drawback of octapeptide derivatives, which is the loss of activity in certain receptors.

### 1.2. Somatostatin Structure

The structure of somatostatin in solution shows a high conformational flexibility [22,23,24,25] and its native structure is now considered as an ensemble of several interchanging conformations in equilibrium [26]. Despite three decades of work in this field, it has not been possible to determine the specific conformation of SRIF that is recognized by each receptor subtype. Nevertheless, the synthesis of shorter ring analogs in the last years has afforded numerous compounds with different receptor selectivity. Their smaller size and reduced mobility has allowed the structural characterization of some of these analogs [13,27,28,29,30,31]. However, these structures cannot provide an accurate picture of the different bioactive conformations of SRIF, due to significant differences in sequence and function between SRIF and the published octapeptide analogs.

Several studies have been devoted to determining the role of the different residues in the natural hormone sequence [32]. It is well accepted that the four amino acid sequence Phe7-Trp8-Lys9-Thr10 constitutes the pharmacophore. Small modifications of these four residues can be made without a significant loss of activity. Examination of the three marketed analogs reveals that all have a similar four residue fragment: Lys9 is always conserved; Trp8 has been replaced by its enantiomer, and Thr by Val. In all cases Phe6 and Phe11 have been replaced by cysteine as a surrogate of the putative Phe6-Phe11 aromatic interaction.

**Figure 3:**
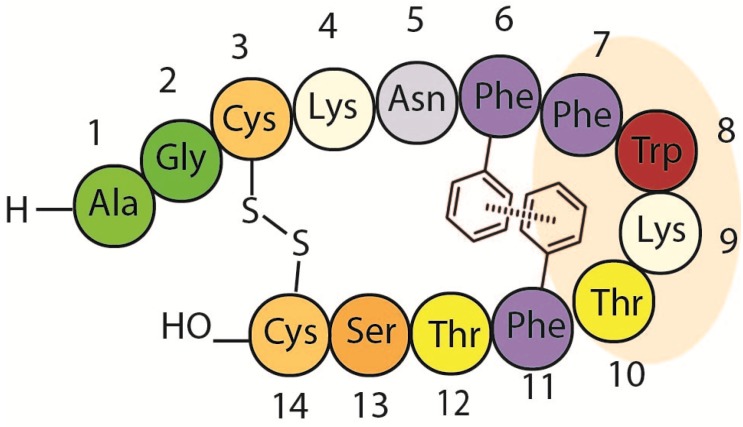
Proposed Phe6-Phe11 aromatic stabilizing interaction.

Early studies [33] uncovered the possibility that an aromatic interaction between Phe6 and Phe11 could play a key role in the structural stabilization of the hormone (Figure 3). This hypothesis was supported by pioneering NMR experiments carried out by Arison *et al*. [34]. The temperature dependence in the chemical shift showed that the Phe6 protons where in the shielding cone of an aromatic ring. NMR studies of both SRIF and a shorter analogue carried out by Cutnell *et al*. [35] provided geometric information about this non-covalent interaction. The temperature effect on the Phe6 protons shifts was not consistent with parallel stacking, so a perpendicular aromatic interaction was proposed (with *o*- and *m*-H of Phe6 pointing near the center of a second aromatic ring). The authors also showed that replacement of Phe7 by Ala in this smaller analogue retained the up-field *o*-H resonance of Phe6, whilst Phe11 substitution with Ala completely eliminates this shift. This observation confirmed the hypothesis that Phe11 is shielding the aromatic ring of Phe6 in this “perpendicular” aromatic interaction, and this interaction is also present in the active conformations of the natural counterpart. However, other authors have re-examined this issue and, no NOE’s between Phe11 and any of the other aromatic rings were detected using 2D NOESY ^1^H-NMR studies [36,37]. Van Binst and co-workers concluded from this that the Phe6-Phe11 interaction proposed by Veber *et al*. was not significantly present in the main conformations of SRIF in aqueous solution.

### 1.3. Somatostatin Analogs with Mesitylalanine

Exploring the putative aromatic-aromatic interactions that stabilize some bioactive conformations in SRIF seemed to be a very straightforward way to generate analogs with enhanced stability, conformational rigidity and different biological activity. The present knowledge about aromatic side-chain interactions, together with the increased interest in the structure-activity relationships, prompted us to exploit the effect of such interactions in new SRIF analogs. As a part of an ongoing study of 14-amino acid SRIF analogs, we decided to incorporate mesitylalanine residues with higher electron density than phenylalanines, and with the intrinsic rigidity provided by the *ortho* substitution [38]. To this end, we synthetized ten peptides containing Msa; four of them were previously described [21], but are included here for completeness (Table 1).

**Table 1:** Somatostatin analogs obtained by replacement of the Phe in positions 6, 7 and/or 11 by mesitylalanine (Msa). In compounds **4**–**6** the natural Trp in position 8 was replaced by D-Trp. 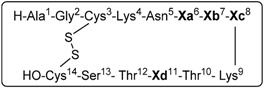

Entry	Xa(6)	Xb(7)	Xc(8)	Xd(11)	Peptide analog
1	**Msa**	Phe	L-Trp	Phe	[L-Msa6]-SRIF, **1**
2	Phe	**Msa**	L-Trp	Phe	[L-Msa7]-SRIF, **2** ^a^
3	Phe	Phe	L-Trp	**Msa**	[L-Msa11]-SRIF, **3**
4	**Msa**	Phe	**D-Trp**	Phe	[L-Msa6,D-Trp8]-SRIF, **4** ^a^
5	Phe	**Msa**	**D-Trp**	Phe	[L-Msa7,D-Trp8]-SRIF, **5** ^a^
6	Phe	Phe	**D-Trp**	**Msa**	[L-Msa11,D-Trp8]-SRIF, **6** ^a^
7	**Msa**	**Msa**	L-Trp	Phe	[L-Msa6,7]-SRIF, **7**
8	**Msa**	Phe	L-Trp	**Msa**	[L-Msa6,11]-SRIF, **8**
9	Phe	**Msa**	L-Trp	**Msa**	[L-Msa7,11]-SRIF, **9**
10	**Msa**	**Msa**	L-Trp	**Msa**	[L-Msa6,7,11]-SRIF, **10**

^a^ Described in the preliminary communication [21].

The first group of compounds **1**–**3** were obtained by the replacement of each of the three phenylalanine residues on the natural 14-amino acid sequence (positions 6, 7 and 11) with Msa. Since it is known that exchanging L-Trp with its enantiomer does not significantly change the activity profile and increases the stability of the molecule [39,40,41] a second group of peptides with D-Trp in position eight was prepared (compounds **4**–**6**). Two replacements of phenylalanine residues with Msa afforded three more peptides **7**–**9**. Finally, the replacement of all three phenylalanine residues with Msa afforded peptide **10**. Fmoc-L-3-mesitylalanine was obtained either following the procedure previously developed by our group [42] or by enantioselective hydrogenation [43,44].

## 2. Results and Discussion

### 2.1. Synthesis of Tetradecapeptides with Mesitylalanine

Peptide analogs **1**–**10** (Table 1) were prepared by solid-phase peptide synthesis (SPPS) on 2-chlorotrityl chloride resin, using the Fmoc/tBu strategy. The coupling of the first amino acid Fmoc-Cys(Trt)-OH was performed in dichloromethane (DCM) in the presence of *N*-ethyldiisopropylamine (DIPEA). After the coupling was finished, the remaining free chlorides were capped with methanol. The next Fmoc-protected amino acids (and the last Boc-Ala-OH) were added using *N*,*N*’-diisopropylcarbodiimide (DIC) and 1-hydroxybenzotriazole (HOBt) in DMF. Piperidine in DMF (20%) was used to remove the Fmoc protecting group. When the non-natural amino acid was incorporated, only 1.5 equiv were used in the coupling. The formation of the disulfide bridge was achieved in solution at room temperature with iodine (I_2_), after cleavage (DCM/TFE/AcOH) of the fully protected linear peptide from the resin. Finally, side chain deprotection using a mixture of TFA/DCM/anisole/H_2_O for 4 h afforded SRIF-14 analogs in modest yields (20%–50%) and moderate purities that were improved up to 99% by HPLC chromatography.

### 2.2. Serum Stability

The low stability of natural SRIF (with a half-life of 2–3 min *in vivo* in human plasma) is one of the main drawbacks of its pharmaceutical use. Thus, we were interested in determining whether our new analogs had longer lifetimes than the wild-type SRIF molecule. To this end, we determined the half-life of the new molecules in human serum and compared them with the values obtained for SRIF, [D-Trp]-SRIF and octreotide. The results of this are shown in Table 2. Peptidic analogs **1**–**3**, containing only one Msa residue, showed low serum stabilities. Among them, only analogue **2** was more stable than SRIF. However, analogs containing the double modification of one Msa residue and D-Trp8 (**4**–**6**) showed a remarkable increase in stability (7 to 20-fold larger than SRIF). Peptides with two Msa residues (compounds **7**, **9**) also showed higher stability than SRIF, with peptide **7** having a half-life of 43.9 h (*versus* 2.75 h of SRIF). Analog **10**, with three Msa residues in its sequence, showed the highest serum stability (34 times more stable than the natural hormone) although, as we will discuss below, this peptide has no affinity toward any of the somatostatin receptors. In summary, the incorporation of non-natural amino acids results in enhanced serum stability for the majority of analogs. The effect of only one residue is relatively small but replacing two or three residues with non-natural amino acids in the somatostatin scaffold increases the overall stability up to 30-fold. Although this value is still far from the stability of octreotide (200 h in serum), the increase from hours to days constitutes a significant improvement.

### 2.3. Binding Activity

The receptor subtype selectivity was measured using competition-binding assays in Chinese hamster ovary (CHO) cell lines. For comparative purposes, the same tests were applied to SRIF-14, [D-Trp8]-SRIF and octreotide. In short, stable CHO cell lines that specifically express each of the five SSTR receptors were cultured and centrifuged to extract the membranes. Inhibitor selectivity was determined with a competitive assay using ^125^I-labeled and unlabeled SRIF-14 in all five receptors. All binding data is shown in Table 2. As can be readily seen, the new peptides show a wide scope of biological activities, ranging from moderately active in all receptors (compound **1**), to remarkably selective (compounds **2**–**5**) or even completely inactive (compound **10**). These affinity values will be discussed below in more detail in parallel with their structures.

**Table 2:** *K*_i_ values (nM) to receptors SSTR1-5 of peptides **1**–**10**, SRIF, [D-Trp8]-SRIF and octreotide.

Peptide	SSTR1	SSTR2	SSTR3	SSTR4	SSTR5	t_1/2_ (h) ^a^
Somatostatin, SRIF	0.43 ± 0.08	0.0016 ± 0.0005	0.53 ± 0.21	0.74 ± 0.07	0.23 ± 0.04	2.75
[D-Trp8]-SRIF	0.32 ± 0.11	0.001 ± 0.0007	0.61 ± 0.02	5.83 ± 0.44	0.46 ± 0.24	19.7
Octreotide	300 ± 85	0.053 ± 0.011	15.2 ± 5.9	>10^3^	11.53 ± 1.91	200
[L-Msa6]-SRIF, **1**	8.52 ± 1.45	1.49 ± 1.45	1.36 ± 1.45	3.62 ± 1.45	0.91 ± 1.45	2.1
[L-Msa7]-SRIF, **2** ^c^	4.17 ± 1.45	0.019 ± 0.009	>10^3^	28.72 ± 6.9	>10^3^	5.2
[L-Msa11]-SRIF, **3**	19.97 ± 5.26	0.024 ± 0.004	2.8 ± 0.22	6.45 ± 2.23	2.1 ± 0.70	1.7
[L-Msa6_D-Trp8]-SRIF, **4** ^c^	3.08 ± 0.9	4.55 ± 0.66	0.78 ± 0.1	4.70 ± 0.92	0.36 ± 0.003	26
[L-Msa7_D-Trp8]-SRIF, **5** ^c^	0.33 ± 0.09	0.0024 ± 0.001	7.49 ± 0.63	>10^3^	>10^3^	25
[L-Msa11_D-Trp8]-SRIF, **6** ^c^	3.35 ± 1.32	0.14 ± 0.06	1.31 ± 0.2	>10^3^	0.73 ± 0.19	41
[L-Msa6,7]-SRIF, **7**	>10^3^	14.69 ± 0.82	>10^3^	>10^3^	>10^3^	43.9
[L-Msa6,11]-SRIF, **8**	>10^3^	>10^3^	13.34 ± 2.92	>10^3^	9.12 ± 0.61	nm ^b^
[L-Msa7,11]-SRIF, **9**	105.75 ± 30.6	1.37 ± 0.32	>10^3^	>10^3^	>10^3^	10
[L-Msa6,7,11]-SRIF, **10**	>10^3^	>10^3^	>10^3^	>10^3^	>10^3^	93.3

*K_i_* values are mean ± SEM. Shaded cells represent data in close proximity to the SRIF values. ^a^ Human serum half-life. ^b^ Not measured. ^c^ Described in the preliminary communication [21].

### 2.4. NMR Structure

#### 2.4.1. Compounds **1**–**3** with One Msa Residue

**Figure 4:**
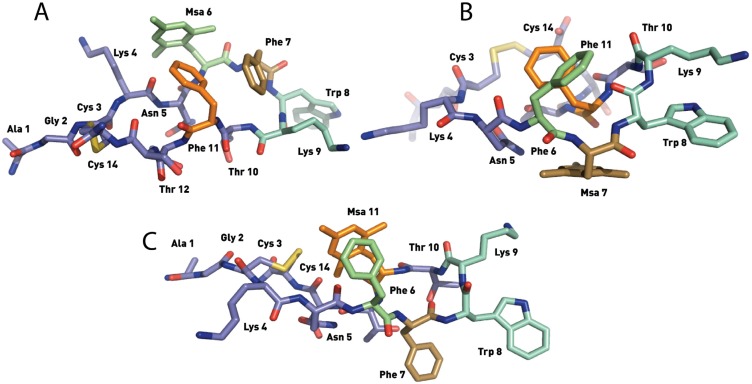
Structures of the lowest energy conformers of [L-Msa6]-SRIF (**1**) (**A**), [L-Msa7]-SRIF (**2**) (**B**) and [L-Msa11]-SRIF (**3**) (**C**). Hydrogen atoms have been omitted for clarity.

**Figure 5:**
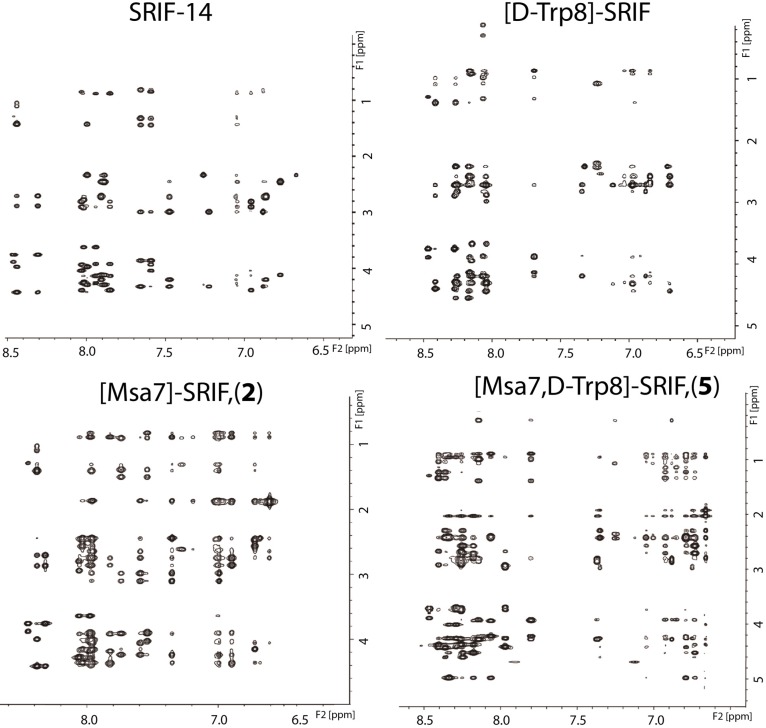
NOESY (600 MHz, D_2_O, 200 ms) of the aromatic ring-long range interaction region of the natural hormone, [D-Trp8]-SRIF, [L-Msa7]-SRIF (**2**) and [L-Msa7,D-Trp8]-SRIF (**5**). NMR data were acquired at 285 K, using trifluoroacetate as a counter-ion at pH 1.5.

**Figure 6:**
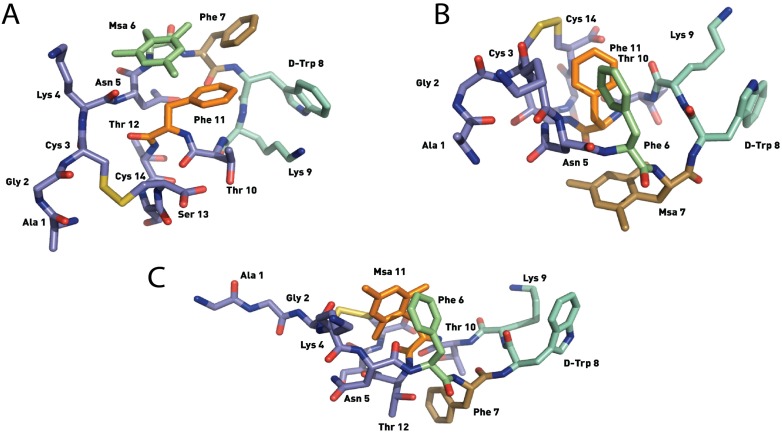
Structures of the lowest energy conformers of [L-Msa6,D-Trp8]-SRIF (**4**) (**A**), [L-Msa7,D-Trp8]-SRIF (**5**) (**B**) and [L-Msa11,D-Trp8]-SRIF (**6**). (**C**). Hydrogen atoms have been omitted for clarity.

The SRIF analogs containing only one Msa insertion (in position 6, 7 or 11, analogs **1**–**3**) were analyzed by NMR. Unlike somatostatin, which populates several conformations in aqueous solution, the two dimensional TOCSY and NOESY homonuclear experiments [45] showed a major set of NOE peaks. The well-defined 2D spectra of compounds **1**–**3** enabled us to characterize their main conformation in solution using the software Crystallography & NMR System (CNS) [46]. To generate the list of experimental restraints required for the calculation, the volume of all assigned peaks was integrated, and then converted into distances. Three calculations (120 structures each) were run until the best match between the NMR assignments and final structures was obtained. Based on these results we concluded that under the experimental conditions used, compounds **1**–**3** were sufficiently structured to obtain defined families of structures that are in good agreement with the experimental data (Figure 4, Figure 5 and Figure 6).

The 3D structure of [L-Msa6]-SRIF (**1**) showed a singular aromatic ring cluster among Msa6, Phe7 and Phe11 as well as a hairpin (Figure 4A). This arrangement of aromatic rings was defined by a number of NOE’s among the aromatic protons of these three residues. However, the absence of an additional stabilizing effect in the hairpin area increased the conformational mobility of this peptide. Thus, our NMR data suggested that other minor conformations were present in solution. As shown in Table 2, compound **1** binds to all receptors although with weaker affinity than somatostatin. Furthermore, the presence of only one unnatural residue in its sequence did not increase its stability in serum; its half-life is slightly lower than that of the natural hormone.

The NMR data of [L-Msa7]-SRIF (**2**) clearly showed that this compound was conformationally more rigid than **1** in solution. The 3D structure of the lower energy conformers showed a highly structured region from residues **6** to **11**, with a clear aromatic interaction between Phe6 and Phe11 (Figure 4B) which can be defined as an *edge-to-face* interaction*.* The Msa residue in the seventh position does not participate in the aromatic interaction, lying flat at the opposite face of the molecule. However, it probably plays an essential role in conformer stabilization, helping the aromatic rings of Phe6 and Phe11 to attain the optimal geometry. NOE crosspeaks of Lys9-Trp8 interaction were weak and difficult to identify. Peptide **2** was highly selective towards receptor SSTR2, with a *K*_i_ of 0.019 nM. Interestingly, and perhaps due to its conformational stability, the stability of peptide **2** in serum (5.2 h) is almost double to that of SRIF, and has the highest stability of the three analogs **1**–**3** with only one residue modification.

The set of low energy conformers calculated for peptide [L-Msa11]-SRIF (**3**) showed a remarkable level of convergence in the majority of geometrical parameters as a result of the high number of experimental restraints observed and the intrinsically high conformational rigidity in the molecule. In this analog, the Msa amino acid at position 11 participates in a π-π aromatic interaction, with the phenyl ring of Phe6 oriented in an *offset-tilted* arrangement on one side of the molecular plane (Figure 4C). Moreover, the Phe7 ring lies on the other side of the molecular plane as it occurs in the structure of molecule **2**, but in **3** the orientation is almost perpendicular to the plane.

As observed in peptide **2**, the interaction between the side chains of Lys9 and Trp8 is instrumental in defining the formation of the hairpin. However, these interactions are not strong enough to fix the L-Trp8 in a fixed rotamer, with some conformations of peptide **3** bringing the L-Trp8 indole side-chain in close proximity to the benzyl side-chain of Phe7. Remarkably, the binding profile of this compound reflects that it is highly selective toward SSTR2, despite having a dissociation constant larger than that of compound **2**. However, its stability in serum is poor, displaying a half-life even shorter than that of natural SRIF.

#### 2.4.2. Compounds **4**–**6** with One Msa Residue and D-Trp8

The discovery of unique conformations in peptides **1**–**3**, which allowed us to determine their 3D structures by NMR in aqueous solution, prompted us to concentrate our efforts on increasing the stability of these analogs while maintaining their binding properties. Pioneering work by Rivier and co-workers in 1975 [39] showed that [D-Trp8]-SRIF exhibits an excellent binding profile toward all receptors but higher stability than its [L-Trp8]-SRIF natural counterpart. Indeed, several studies [39,40] have demonstrated that the Lys9 side chain is more effectively shielded by [D-Trp8] because the D-configuration of tryptophan favors an orientation where the indole ring is in close proximity to the aliphatic side chain of Lys9. These contacts not only maintain the Lys9 side chain in a defined orientation, but also induce the formation of the hairpin centered at Trp8-Lys9.

Our aim was to investigate whether the introduction of the [D-Trp8] residue would increase the stability of the peptides containing Msa side-chains while maintaining the conformational structure and the binding profile. Therefore, we prepared three new analogs **4**–**6** carrying Msa and [D-Trp8] residues by SPPS and determined whether these substitutions affected either the structure or stability of the peptides. In addition, we also prepared the [D-Trp8]-SRIF analog and compared its NMR properties with that of the natural [L-Trp8]-SRIF. A section of the 2D NOESY spectra of the natural hormone, [D-Trp8]-SRIF, [L-Msa7]-SRIF (**2**) and [L-Msa7,D-Trp8]-SRIF (**5**), is shown in Figure 5 to illustrate the improvement in the NMR data. As it can be observed, [D-Trp8]-SRIF maintains the intrinsic flexibility of the natural hormone, whereas **2** and **5** show an increasing amount of NOE signals.

The conformational flexibility of both SRIF and [D-Trp8]-SRIF accounts perfectly for its functional versatility against all receptors (SSTR1-5) (Table 2). In both cases, the coexistence of several different conformations prevented us from carrying out definitive structural studies. In contrast, the 2D spectra of compounds **4**–**6** were extremely well-defined, enabling us to characterize their main conformation in solution from the NMR restraints and using the CNS software. As before, the volume of all assigned peaks was integrated, transformed into distances and used to generate the list of experimental restraints for calculation. Three sets of calculations (120 structures each) were run until the best match between assignments and final structures was obtained. As expected, the generated structures (from the experimental NMR data) of these new peptides showed a clear increase in convergence not only with respect to SRIF and [D-Trp8]-SRIF, but also with respect to monosubstituted analogs **1**–**3**.

Peptide [L-Msa6,D-Trp8]-SRIF (**4**) gave a well-defined conformation in solution (Figure 6A) in which the two aromatic rings of Msa6 and Phe11 are markedly proximal due to the enhanced aromatic interaction, while Phe7 is also participating in the aromatic cluster. This cluster of three aromatic rings is similar to the one found in [L-Msa6]-SRIF (**1**). It is apparent that the region containing residues **5**–**11** is much more structured than the rest of the molecule. The pharmacophore region of the most stable conformations matched perfectly (residues **6** to **11**). On the other hand, the rest of the molecule is less rigid. The binding profile of peptide **4** is also similar to peptide **1**, although its affinity towards receptors SSTR3 and SSTR5 is comparable to that of the natural hormone, with an affinity 20–30 times more potent than octreotide (Table 2). The lack of affinity of both peptides **1** and **4** towards receptor SSTR2 (which both have a Msa residue in the sixth position) does not support the idea that residue **6** interacts via a π-donation with an amino acid side chain in SSTR2, as suggested in Hirschmann’s hypothesis [47].

**Figure 7:**
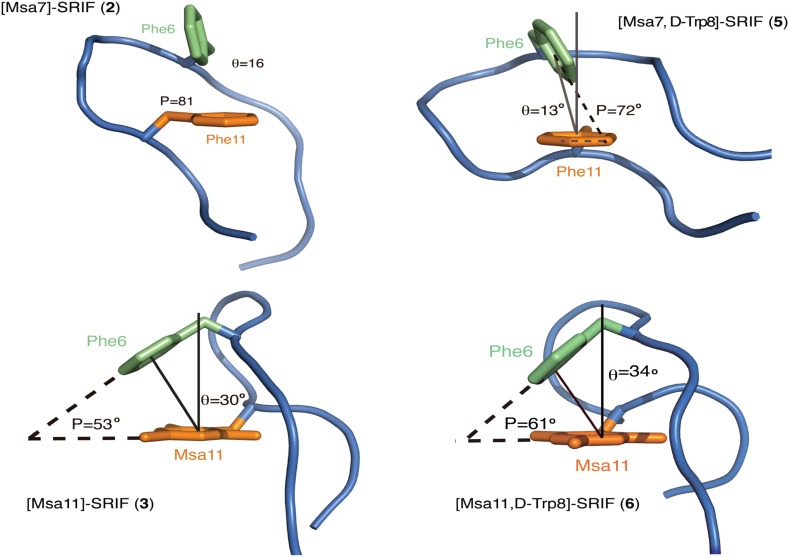
Schematic representation of the aromatic interactions in peptides **2**, **3**, **5** and **6**.

Peptide [L-Msa7,D-Trp8]-SRIF (**5**) also showed a strong interaction between Phe6-Phe11 and a well-defined hairpin at the pharmacophore region (Figure 6B). This hairpin was also observed in the [L-Msa7]-SRIF peptide (**2**). Again, the calculated structures display a good degree of convergence, being the pharmacophore region (residues **5**–**11**) more structured than the remaining part of the molecule. In contrast to peptide **2**, a large set of interactions between Trp8 and Lys9 can be clearly observed in compound **5**. In this case, the Lys-Trp interaction is reflected in the upfield shift γ protons of Lys9 which are shielded by the aromatic indole ring. Furthermore, the aromatic-aromatic interactions between residues Phe6 and Phe11 in peptides **2** and **5** are very similar as depicted in Figure 7, (the backbone is shown as a cartoon representation and the Phe6 and Phe11 residues as sticks). The molecules were rotated with respect to previous figures to provide a close view of these interactions. The aromatic pair association can be classified as an *edge-to-face* type [48,49]. The shortest inter-residue carbon-carbon distances (SICD) are 3.3 and 2.7 Å respectively. The angle formed between the ring centroid to centroid segment and the z-axis of an axial system centered on the centroid of the reference ring (θ) is very small (16° and 13° for **2** and **5** respectively). The ring planes are almost perpendicular with interplanar angles (P) of 81° and 72° respectively. The mesityl ring in peptide **5**, as in peptide **2**, is not involved in any aromatic interactions. The aromatic ring is lying flat at the other side of the molecule, probably facilitating the interaction between both Phe6 and Phe11 by steric repulsion. The lowest energy conformation is fairly similar to **2**, but the synergistic stabilizing effect due to the presence of the D-Trp8 leads to the least flexible 14-residue SRIF analogue described to date. Its outstanding affinity for SSTR2 probably correlates with having a well-defined structure close to the conformation that fits best in the structure of the SSTR2 receptor, which is so far uncharacterized. Its inhibition constant was 22-fold lower than that of octreotide and similar to SRIF. Peptide **5**, unlike octreotide, also exhibited an impressive affinity toward SSTR1, at the same level than SRIF.

The analogue [L-Msa11,D-Trp8]-SRIF (**6**) also had remarkable conformational stability. The most stable conformers showed a strong π-π aromatic interaction between the Phe6 and Msa11 residues as displayed in Figure 6C. It was also apparent that peptides **3** and **6** showed a remarkable similarity to one another. The geometric values of the aromatic interaction in peptides **3** and **6** are shown in Figure 7 showing only the backbone and the two aromatic rings of the most stable conformer. In this case, the position of the two aromatic rings fits as an *offset-tilted* interaction, with the θ angles wider than in previous analogs (30° and 34° for **3** and **6** respectively). In this case, the interplanar angles (P) are 53° and 61° (Figure 7). Peptide **6** showed high affinity towards the SSTR5 receptor, although it displayed a lower level of selectivity, since it also binds to SSTR1, SSTR2 and SSTR3 receptors with comparable affinities. Its stability in serum is very high (41 h), which is higher than any of the analogs prepared carrying one Msa and the D-Trp8 residues.

Overall, the structures of peptides with L-Trp8 (compounds **1**–**3**) are remarkably similar to those with D-Trp8 (compounds **4**–**6**). Nearly all of the observed NOE’s for compounds **1**–**3** were also present in the spectra of compounds **4**–**6**. This suggests that in peptides **4**–**6**, the D-Trp8 residue shifts the equilibrium toward conformations that are already populated in a considerable manner in peptides **1**–**3**. The NMR data of compounds **4**–**6** showed a higher increase in number and intensity of the contacts between Trp8-Lys9 than peptides **1**–**3**. Furthermore, the contrast between the γ protons of Lys9 in peptides **1**–**3** (which showed a similar shift to those in [L-Trp8] SRIF), and analogs **4**–**6** (which were significantly upfield shifted) could be attributed to the efficient interaction of the Lys9 and D-Trp8 side chains.

#### 2.4.3. Compounds **7**–**10** with Multiple Incorporations of Msa Residues

Since a simple substitution of Phe by Msa in the natural sequence remarkably increased the conformational rigidity of the analogs, we went a step further and studied whether the addition of multiple Msa residues could have an impact on their structure and/or their interaction with the receptors. To obtain this information, we prepared peptides **7**–**10** (Table 1) using the general SPPS methods. Analogs **7**–**10** showed a minimal number of NOE signals (mainly sequential NOE’s could be assigned), proving that the presence of more than one Msa in the 14-amino acid sequence does not improve the conformational stability of these SRIF derivatives in solution. A probable explanation for this negative effect on the fold of the peptide can be rationalized based on the large steric hindrance imposed by the ortho- methyls of Msa residues, preventing the aromatic interaction between Msa residues. This explanation is supported by the lack of NOEs between the aromatic mesityl protons. It appears that the 14-residue-sequence of somatostatin would accommodate more than one Msa in positions 6, 7 and/or 11, but this is at the price of losing the structural properties of the native hormone. Most importantly, the presence of several Msa residues reduces the affinity of the peptides towards the five SSTR receptors. In particular, compound **10**, containing three Msa residues, was completely inactive towards all of the SSTR receptors (Table 2).

## 3. Experimental

### 3.1. General Syntheses of Peptides

The syntheses were performed by SPPS on a 2-Cl-Trt resin (1.60 mmol/g) using the Fmoc/tBu strategy. Initially, the first amino acid Fmoc-L-Cys(Trt)-OH (1 equiv) was coupled in the presence of DIPEA (4 equiv) in DCM as solvent for 40 min and finally end-capped with methanol (0.8 mL g^−1^). Then, Fmoc removal was performed by treating the peptidyl resin with 20% piperidine in DMF (1 × 1’ and 1 × 5’). The second amino acid Fmoc-L-Ser(tBu)-OH (2.5 equiv) was coupled using DIC (2.5 equiv) and HOBt (2.5 equiv), as activating reagents, in DMF for 40–60 min. Kaiser test was used to check coupling completions [50]. This procedure was repeated for the following eleven Fmoc-protected amino acids and for the last Boc-L-Ala-OH. When Fmoc-L-Msa-OH was coupled, only 1.5 equiv were used. The cleavage of the fully protected linear peptide from the resin was carried out using a DCM/TFE/AcOH mixture for 2 h. The formation of the disulfide bridge in all the analogs was achieved using iodine at room temperature and then quenched with an aqueous solution of sodium thiosulphate 1N. The aqueous layer was extracted with DCM (3 × 4 mL), the combined organic layer was washed with a mixture of an aqueous citric acid 5% solution/sodium chloride (1:1) and evaporated under reduced pressure. Finally, total deprotection of the side-chains was performed using an acidic mixture (TFA/DCM/anisole/H_2_O 12/6/2/1). Then, the remaining solution was washed with heptane (20 mL) and the aqueous layer was precipitated in Et_2_O (−10 °C) afforded somatostatin analogs. Analogue [D-Trp8]-SRIF, used for receptor binding assays and NMR data comparison, was synthesized following this general procedure by incorporating Fmoc-D-Trp-OH instead of the natural enantiomer. Analytical and preparative HPLC were carried out with C8 Kromasil columns using gradients of solvent A (0.1% TFA in H_2_O) and solvent B (0.07% TFA in CH_3_CN). Yields of peptides are based in re-calculated loading of the resin.

*[L-Msa6]-SRIF* (**1**): Somatostatin analogue **1** was synthesized following the general procedure from 0.136 g of 2-Cl-Trt resin (1.60 mmol/g) and using Fmoc-L-Msa-OH, affording 0.20 g of **7** in 60% yield (95% purity after purification). HPLC: t_R_ = 14.3 [Gradient 25%–60%B in 20 min, flux: 1 mL min^−1^, λ = 220 nm]. HRMS: calcd for 1678.8; found, 1680.2.

*[L-Msa7]-SRIF* (**2**), *[L-Msa6,D-Trp8-SRIF]* (**4**), *[L-Msa7,D-Trp8-SRIF]* (**5**) and *[L-Msa11,D-Trp8]-SRIF* (**6**) were synthetized according to ref. [21].

*[L-Msa11]-SRIF* (**3**): Somatostatin analogue **3** was synthesized following the general procedure from 0.136 g of 2-Cl-Trt resin (1.60 mmol/g) and using Fmoc-L-Msa-OH, affording 0.28 g of **9** in 44% yield (99% purity after purification). HPLC: t_R_ = 14.1 [Gradient 25%–60%B in 20 min, flux: 1 mL min^−1^, λ = 220 nm]. HRMS: calcd for 1678.8; found, 1680.0.

*[L-Msa6,7]-SRIF* (**7**): Somatostatin analogue **7** was synthesized following the general procedure from 0.10 g of 2-Cl-Trt resin (1.60 mmol/g) and using Fmoc-L-Msa-OH, affording 0.12 g of **10** in 20% yield (98% purity after purification). HPLC: t_R_ = 16.5 [Gradient 25%–60%B in 20 min, flux: 1 mL min^−1^, λ = 220 nm]. HRMS: calcd for 1721.1; found, 1721.0.

*[L-Msa6,11]-SRIF* (**8**): Somatostatin analogue **8** was synthesized following the general procedure from 0.136 g of 2-Cl-Trt resin (1.60 mmol/g) and using Fmoc-L-Msa-OH, affording 0.28 g of **11** in 44% yield (99% purity after purification). HPLC: t_R_ = 15.4 [Gradient 25%–60%B in 20 min, flux: 1 mL min^−1^, λ = 220 nm]. HRMS: calcd for 1721.1; found, 1720.8.

*[L-Msa7,11]-SRIF* (**9**): Somatostatin analogue **9** was synthesized following the general procedure from 0.10 g of 2-Cl-Trt resin (1.60 mmol/g) and using Fmoc-L-Msa-OH, affording 0.13 g of **12** in 44% yield (99% purity after purification). HPLC: t_R_ = 16.4 [Gradient 25%–60%B in 20min, flux: 1 mL min^−1^, λ = 220 nm]. HRMS: calcd for 1721.1; found, 1721.0.

*[L-Msa6,7,11]-SRIF* (**10**): Somatostatin analogue **10** was synthesized following the general procedure from 0.10 g of 2-Cl-Trt resin (1.60 mmol/g) and using Fmoc-L-Msa-OH, affording 0.10 g of **13** in 44% yield (95% purity after purification). HPLC: t_R_ = 18.4 [Gradient 25%–60%B in 20 min, flux: 1 mL min^−1^, λ = 220 nm]. HRMS: calcd for 1762.9; found, 1763.2.

### 3.2. NMR and Computational Methods

NMR experiments were recorded in H_2_O/D_2_O (90/10) without buffer. The resulting solutions had a pH of 1.5–2. The concentration was 5 mM aprox. (4 mg in 0.5 mL). 2D TOCSY and NOESY homonuclear experiments were acquired in a Bruker Avance III spectrometer (600 MHz). Chemical shifts are recorded in ppm. All NMR data was processed with NMRPipe [51] Cara [52] was used to assign the spectra. Distance restraints derived from fully assigned peaks in NOESY experiments were used for structure calculation. The structures were calculated with the programs CNS [46] and StructCalc (StructCalc program was used by courtesy of its recent developers: Pau Martín-Malpartida and Maria J. Macias; unpublished data). Statistics from the analyses are shown in Supporting Information. PyMOL was used to visualize the structures and generate the figures.

### 3.3. Preparation of Cells Stably Expressing the SRIF-14 Receptor

CHO-K1 cells (American Type Culture Collection, Rockville, MD, USA) were maintained in Kaighn’s modification of Ham’s F12 medium (F12K) supplemented with 10% fetal bovine serum. pcDNA3 vectors encoding each of the SSTR receptors were obtained from UMR cDNA Resource Center (University of Missouri, Columbia, MO, USA). CHO-K1 cells were stably transfected with these vectors by using Lipofectamine (Life Tecnologies Corporation, 5791 Van Allen Way, Carlsbad, CA, USA). Stable clones were selected in F12K containing G418 (700 µg/mL) and were screened for SRIF-14 receptor expression and then maintained in a G418 (400 µg/mL)-containing medium. Expression was detected by RT-PCR and western-blot and confirmed by radio ligand binding assays.

### 3.4. Receptor Ligand-Binding Assay

All receptor-binding assays were performed with membranes isolated from CHO-K1 cells expressing the cloned human SRIF-14 receptor, as reported previously [53]. The assay buffer consisted of 50 mM Tris (pH 7.5) with 1 nM EGTA, 5 mM MgCl_2_, leupeptin (10 µg/mL), pepstatin (10 µg/mL), bacitracin (200 µg/mL), aprotinin (0.5 µg/mL) and 0.2% BSA. CHO-K1 cell membranes, radiolabeled SRIF-14 and unlabeled test compounds were diluted in this assay buffer. All assays were performed in 96-well polypropylene plates. Ten micrograms of membrane proteins were incubated with 0.1 nM of ^125^I-Tyr11-SRIF (specific activity 2,000 Ci/mmol) in the presence or absence of various concentrations of unlabeled peptides (1 pM–1,000 nM) in a total volume of 200 µL, for 1 h at 30 °C. The binding reaction was terminated by vacuum filtration over Whatman GF/F glass fibre filters previously pre-soaked in 0.5% (w/v) polyethyleneimine and 0.2% bovine serum albumin, using a 98-well harvester (Inotech Biosystems International Inc., Dietikon, Switzerland). The filters were washed with ice-cold 50 mM Tris-HCl (pH 7.5) and dried, after which scintillator sheets were melted onto the filter and the bound radioactivity was analyzed in a liquid scintillation counter (microβ plus, PerkinElmer, Wallac, Waltham Massachusetts USA). Specific binding was defined as the total ^125^I-Tyr11-SRIF binding minus the amount bound in the presence of 1,000 nM SRIF (non-specific binding). Inhibition curves were analyzed and IC_50_ values were calculated using a curve-fitting program (GraphPad Prism, La Jolla, CA, USA). The *K*_i_ values for the compounds were determined as described by Cheng and Prusoff [54]. Data are the mean ± S.E.M. of at least three separate experiments, each performed in triplicate.

### 3.5. Serum Stability Assay

A peptide solution of 6 mg/mL in water (1.8 mg in 300 µL) was sterilized by filtration (0.22 µm filter). Aliquots from this solution (10 µL) were added to 90 µL of serum (from human male AB plasma, sterile filtered; Sigma-Aldrich, Inc., St. Louis, MO, USA). These solutions were incubated at 35 °C and samples were taken at 0 min, 1 h, 7 h, 17 h, 24 h and 48 h. Each sample was treated with acetonitrile (200 µL) and cooled to 0 °C for 30 min to precipitate the proteins. The suspensions were centrifuged at 10,000 rpm, during 10 min at 4 °C. The process was repeated twice. The solution was filtered through 0.45 µm of PVDF. The samples were analyzed by HPLC; eluent: 20%–80% B (B = 0.07% TFA in acetonitrile); 20 min gradient; flow: 1 mL min^−1^. For each peptide the experiment was repeated twice. Cleavage of the Ala1-Gly2 moiety was not considered as peptide deterioration. The half-life of the peptide in serum was calculated from the analysis of these degradation data. The concentration of the peptide was taken from the chromatogram integration.

## 4. Conclusions

We have synthesized and studied ten new somatostatin analogs (Table 1) that can be grouped as follows: (a) introduction of one Msa residue in positions 6, 7 or 11 of the natural 14 amino acid sequence (peptides **1**–**3**); (b) the same as in (a) but replacing natural L-Trp with D-Trp at position 8 (peptides **4**–**6**); and (c) multiple incorporations of Msa in positions 6, 7 and 11 (peptides **7**–**10**). All analogs were tested for their ability to bind to the SSTR receptors, for their stability in serum (Table 2) and were studied in detail by NMR.

The presence of favorable defined conformations in peptides **1**–**6** allowed us to characterize their structure in detail by computational techniques using NMR restraints. Analogs containing one Msa and D-Trp are more structured than those with the natural amino acids although the most stable set of conformers did not appear to change significantly with the replacement of L-Trp with D-Trp (comparing compounds **1** to **4**, **2** to **5** and **3** to **6**). The increase of conformational rigidity stems from the reinforcement of aromatic interaction between certain residues when Msa is present, and the preferred interaction of the indole ring with the lysine side chain when the Trp8 residue has a D configuration. It is known that Lys9 is more effectively shielded by Trp8 when Trp8 has a D configuration due to the closer proximity between the indole group of Trp and the aliphatic side chain of Lys9. The single modification of introducing one Msa residue combined with the presence of D-Trp8 enhanced what were before weak interactions among the aromatic side-chains in SRIF, leading to analogs with greater stability which possessed higher affinities and selectivity towards the SSTR receptors.

It is worth noting that the majority of the contacts present in the NOESY spectra of these compounds were also detected in the parent compound, indicating that these Msa containing analogs preferentially populates a dominant conformation that already exists in solution in somatostatin. In all cases (compounds **1**–**6**) we have found that non-covalent aromatic interactions occur between residues in sixth and eleventh position. Therefore our results are consistent with the hypothesis described by Veber and co-workers [12] that an aromatic interaction between Phe6 and Phe11 stabilizes the bioactive conformation to a certain degree in SRIF. Analogs **2** and **5** satisfy both high conformational rigidity and an excellent SSTR2 selectivity; consequently, the 3D structure of these 14-aa SRIF analogs should be close to that of SRIF binding receptor SSTR2.

The structures of peptides with L-Trp8 (compounds **1**–**3**) are remarkably similar to those with D-Trp8 (compounds **4**–**6**). It appears that the configuration of this amino acid does not play a fundamental role in the architecture of the peptide. However, the presence of D-Trp8 proved to be important not only in hairpin stabilization, but also to increase the serum stability. This could be due to the increased stability to proteases of peptides with D-residues and also to the increased overall conformational stability of the peptide. The combined presence of both Msa and D-Trp8 (compounds **4**–**6**) could be a synergistic effect and explains the higher serum stability of peptides **4**–**6** than [D-Trp8]-SRIF or compounds **1**–**3**. The enrichment of non-covalent interactions (stabilization of the hairpin area due to the presence of D-Trp8 and intensification of the aromatic-aromatic interactions due to the Msa) explains their higher serum stability in comparison to SRIF. However, peptides **7**–**10** with two or three Msa residues, although possessing a longer half-life in serum, do not show any defined structure in solution (their 3D structure could not be determined) and have poor affinities towards the majority of SSTR receptors.

The potential therapeutic utility of SSTR-selective ligands has been extensively reported [9,10,11,12]. In the past decade, the somatostatin-based receptor-targeted anti-cancer therapy has emerged as a promising tool in order to improve the traditional chemotherapy [55,56]. Precisely, SSTR2 is expressed at remarkably higher levels in many tumor cells relative to normal tissues. The coupling of potent chemotherapeutic agents to SSTR2-selective somatostatin analogs has provided new cytotoxic SRIF-conjugates that selectively target SSTR2-specific sites, displaying significant anti-tumor abilities in many different types of tumors [57,58]. Due to the short half-life of full length somatostatin analogs, only octreotide derivatives are currently used in SSTR-targeted chemotherapy. In this regard, an array of additional investigations of the use of peptide **5** (10 times more stable in serum than SRIF and 10-fold more active against SSTR2 than octreotide) in receptor-targeted anti-cancer therapy is currently underway in our laboratory.

In summary, the replacement of Phe residues by Msa in the original somatostatin sequences has allowed us to fine-tune the structural and biological properties of the corresponding peptide analogs, facilitating deeper insights to the main factors that control the conformation and receptor selectivity of this important hormone.
